# Impact of a post-prescription audit and feedback antimicrobial stewardship intervention on inappropriate carbapenem prescribing: an interrupted time series analysis

**DOI:** 10.1093/jacamr/dlaf236

**Published:** 2025-12-03

**Authors:** Flavio Sangiorgi, Pierluigi Del Vecchio, Eugenia Magrini, Emanuele Rando, Beatrice Liguoro, Alessia Frater, Francesca Giovannenze, Massimo Fantoni, Carlo Torti, Rita Murri

**Affiliations:** Dipartimento di Sicurezza e Bioetica, Università Cattolica del Sacro Cuore, Rome, Italy; UOC Malattie Infettive ad Interesse Chirurgico, Clinical Infectious Diseases Department, National Institute for Infectious Diseases Lazzaro Spallanzani IRCCS, Rome, Italy; UOC Malattie Infettive—Dipartimento Scienze Mediche e Chirurgiche, Fondazione Policlinico Universitario A. Gemelli IRCCS, Rome, Italy; Dipartimento di Sicurezza e Bioetica, Università Cattolica del Sacro Cuore, Rome, Italy; Dipartimento di Sicurezza e Bioetica, Università Cattolica del Sacro Cuore, Rome, Italy; Unidad Clínica de Enfermedades Infecciosas y Microbiología, Hospital Universitario Virgen Macarena, Departamento de Medicina, Facultad de Medicina, Universidad de Sevilla, Instituto de Biomedicina de Sevilla (IBiS)/CSIC, Seville, Spain; Centro de Investigación Biomédica en Red de Enfermedades Infecciosas (CIBERINFEC), Madrid, Spain; Dipartimento di Sicurezza e Bioetica, Università Cattolica del Sacro Cuore, Rome, Italy; Dipartimento di Sicurezza e Bioetica, Università Cattolica del Sacro Cuore, Rome, Italy; UOC Malattie Infettive—Dipartimento Scienze Mediche e Chirurgiche, Fondazione Policlinico Universitario A. Gemelli IRCCS, Rome, Italy; UOC Malattie Infettive—Dipartimento Scienze Mediche e Chirurgiche, Fondazione Policlinico Universitario A. Gemelli IRCCS, Rome, Italy; Dipartimento di Sicurezza e Bioetica, Università Cattolica del Sacro Cuore, Rome, Italy; UOC Malattie Infettive—Dipartimento Scienze Mediche e Chirurgiche, Fondazione Policlinico Universitario A. Gemelli IRCCS, Rome, Italy; Dipartimento di Sicurezza e Bioetica, Università Cattolica del Sacro Cuore, Rome, Italy; UOC Malattie Infettive—Dipartimento Scienze Mediche e Chirurgiche, Fondazione Policlinico Universitario A. Gemelli IRCCS, Rome, Italy

## Abstract

**Background:**

Antimicrobial stewardship programs (ASPs) are essential to improve antibiotic prescribing. This study evaluated the impact of a post-prescription audit and feedback intervention on carbapenem prescribing appropriateness in a large university hospital.

**Methods:**

This retrospective observational study utilized interrupted time series (ITS) analysis, employing an Autoregressive Integrated Moving Average (ARIMA) model, to assess carbapenem prescribing across three consecutive phases: a 12-month pre-intervention, a 6-month intervention, and a 6-month post-intervention follow-up. Carbapenem prescribing appropriateness was retrospectively evaluated using an in-house developed algorithm, based on international and national guidelines and institutional protocols. The intervention involved bedside consultations by infectious diseases specialists employing a post-prescription audit with face-to-face feedback.

**Results:**

We evaluated 1825 carbapenem therapies, primarily prescribed for suspected/confirmed bloodstream infections (46%, 843/1825). Among these, 458 (25%) were deemed inappropriate, mainly due to unnecessarily broad-spectrum use (72%, 331/458). The ITS-ARIMA model showed an immediate 11% reduction in the rate of inappropriate prescriptions during the first month of intervention phase (*P* = 0.001), followed by a non-significant downward trend during the remaining intervention period. However, an immediate 14.9% increase in inappropriate prescriptions was observed at the onset of the post-intervention phase (*P* = 0.001), indicating a rebound effect after the withdrawal of the active stewardship intervention.

**Conclusions:**

Implementing a post-prescription audit and face-to-face feedback intervention was associated with a short-term improvement in carbapenem prescribing appropriateness. We observed a reduction in the trend of inappropriateness, although this change was not statistically significant. Future studies should investigate strategies for implementing sustainable ASPs optimizing human resources and time investment.

## Introduction

Antimicrobial resistance (AMR) poses a significant global challenge to modern medicine. Each year in the European Union (EU), over 35 000 deaths are attributed to infections caused by antimicrobial-resistant bacteria.^1^

Antimicrobial stewardship (AMS) involves a coordinated set of interventions designed to monitor and optimize antibiotic prescription and use by both healthcare professionals and patients. When AMS is implemented in conjunction with Infection Prevention and Control programmes helps to reduce the spread of multidrug-resistant organisms (MDROs).^2^ These measures require coordination at global, national and local levels, aligned with the ‘One Health’ approach.^3^ Among AMS strategies, post-prescription audit and feedback interventions have shown consistent benefits across different healthcare settings, improving the appropriateness of therapy, reducing unnecessary antibiotic exposure and associated adverse effects, and supporting prescribers’ decision-making.^4–7^

However, defining ‘appropriate’ versus ‘inappropriate’ antibiotic therapy remains challenging, as no universal consensus exists on appropriateness criteria, particularly for empirical therapy.^8^ While a definition of appropriate antibiotic therapy commonly relies on expert opinion, which considers critical aspects of the prescribing process (e.g. diagnostic pathway, route of administration, drug interactions, and allergies), this approach is inherently subjective and may lack reproducibility outside individual centres. To mitigate the subjectivity of expert-opinion-based methods, some authors have developed computerized antibiotic prescription tools.^9^ These tools define appropriateness according to local guidelines or, in the absence of approved guidelines, based on review and approval by an infectious disease specialist using standardized coding for evaluating antibiotic prescriptions.^10^ Local institutional guidelines are therefore essential to ensure a consistent and objective distinction between appropriate and inappropriate empirical therapy. Carbapenems are frequently used as empirical broad-spectrum agents—often initiated before microbiological results are available and continued without timely de-escalation or for prolonged durations—in many tertiary-care hospitals, including ours. A pattern reflected in recent studies reporting low rates of de-escalation and extended courses that contribute to selective pressure for carbapenem-resistant organisms.^11–14^ These evidences make carbapenems a high-priority target for post-prescription audit and feedback interventions.

The aim of this study is to evaluate the impact of a post-prescription audit and feedback intervention, supported by a computerized algorithm, on the appropriateness of carbapenem prescribing in surgical and medical wards of a large tertiary-care academic hospital.

## Materials and methods

We performed a retrospective observational study using interrupted time series (ITS) analysis at a 1500-bed academic hospital in Rome from February 2022 to February 2024. The study evaluated the appropriateness of carbapenem therapy before, during, and after an AMS post-prescription audit intervention. All carbapenem prescriptions issued in adult medical and surgical wards under the supervision of the AMS Team were included. Wards excluded from the analysis—the Emergency Department, Hematology, Intensive Care Unit, Gynecology, Obstetrics, and Pediatrics—were not under stewardship oversight and managed antimicrobial prescriptions autonomously or through other Infectious Diseases consultants. No further exclusion criteria were applied beyond the ward-based restriction.

The appropriateness of the therapies was assessed based on international and national guidelines, as well as local empirical therapy protocols, tailored to the specific clinical cases.^15–19^ The inappropriateness of antibiotic therapy with carbapenems was considered the primary outcome.

**Figure 1.**
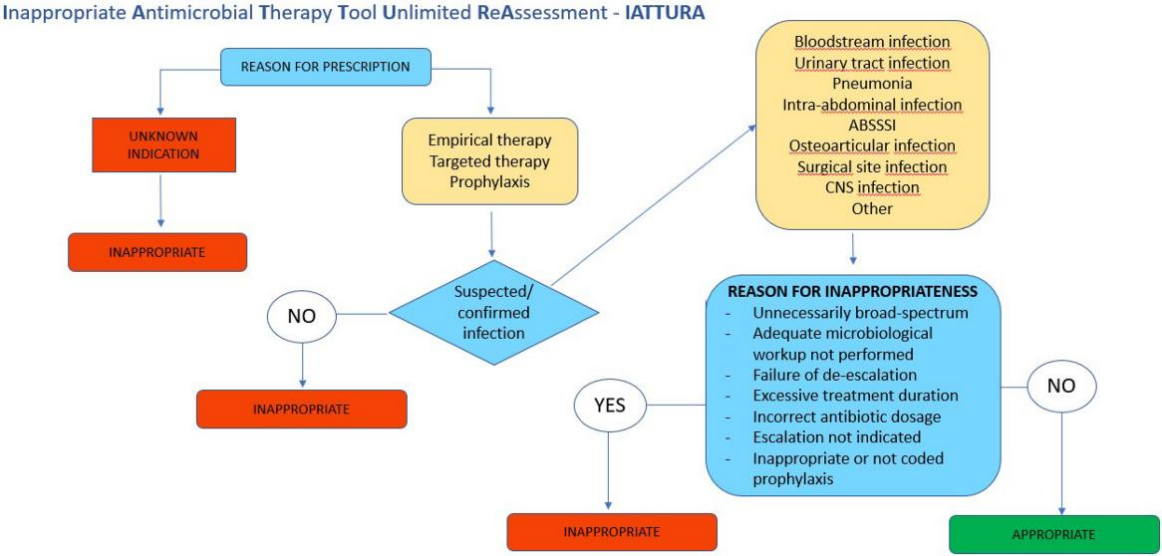
Flow Diagram of the Antibiotic Appropriateness Assessment Logic within the IATTURA Tool. ABSSI, Acute Bacterial Skin and Skin Structure Infections; CNS, Central Nervous System.

To standardize the assessment of antibiotic therapies administered at our hospital, we developed a dedicated computer tool: the Inappropriate Antimicrobial Therapy Tool Unlimited ReAssessment (IATTURA). This tool was designed taking inspiration from the Hospital National Antimicrobial Prescribing Survey (Hospital NAPS) methodology.^20^ Its decision rules were previously reviewed during an internal institutional audit of antibiotic prescribing, which informed refinement of the criteria used in this study. Although no formal inter-rater reliability or diagnostic validation was conducted, the tool’s structure reflects internationally endorsed stewardship principles and locally approved empirical therapy protocols. IATTURA functions as a structured guide for physician review, the detailed decision-making pathway for antibiotic appropriateness within IATTURA is outlined in Figure 1. The eight key variables extracted from the electronic health record are:

1. Antibiotic allergies.
2. Relevant microbiological isolations during hospitalization.
3. Use of anti-Gram-negative beta-lactams in the past 90 days.
4. Antibiotic prescription (specifying drug type and duration).
5. Prescribing physician.
6. Type of infection.
7. Reason for prescription: unknown indication, empirical therapy for unlikely/questionable infection, empirical therapy for probable/possible infection, targeted therapy, or surgical prophylaxis.
8. Reasons for inappropriateness: no indication for antibiotic therapy/colonization only, too broad spectrum, failure of de-escalation, escalation not indicated, failure to perform adequate microbiological workup, incorrect dosage, excessive duration, prophylaxis not appropriate or not coded for the type of procedure.

The intervention was designed and implemented as part of the AMS team’s projects, in alignment with the Joint Commission International Accreditation goals of Fondazione Policlinico Universitario Agostino Gemelli IRCCS. During the intervention phase, antimicrobial prescriptions were reviewed in real time by an infectious diseases (ID) specialist or senior trainee. When a therapy was identified as inappropriate, the prescriber was directly approached for a brief, face-to-face discussion. These interactions, typically occurring within 24 h of prescription review, aimed to explain the reasons for inappropriateness and encourage an appropriate therapeutic modification (de-escalation, discontinuation, or dose adjustment). Feedback was always provided in person by the same ID specialist or trainee who had conducted the review, ensuring a consistent and educational approach. The process maintained a constructive and non-punitive environment, safeguarding the prescriber’s decision-making autonomy. An intervention was considered effective if a therapeutic change occurred, and ineffective if the therapy continued unchanged. When the prescribing physician was reticent, a formal assessment by the ward’s ID consultant could be requested.

Subsequently, a team comprising four ID trainees and one ID specialist retrospectively evaluated the appropriateness of carbapenem prescriptions from two additional periods—the 12 months before and the 6 months after the intervention—to assess the temporal impact of the real-time stewardship program.

### Statistical analysis

Initially, we performed a descriptive analysis to compare the characteristics of antibiotic prescriptions across the three distinct time periods: pre-intervention, intervention, and post-intervention phases. Categorical variables were summarized as absolute and relative frequencies, with differences compared using Fisher's exact test. Continuous or ordinal variables were summarized using medians and interquartile ranges and compared using the Kruskal-Wallis’s test. A *P*-value < 0.05 was considered statistically significant.

After this initial exploratory analysis, we visualized the monthly time series of the proportion of inappropriate carbapenem prescriptions to identify underlying trends, potential seasonality, and outliers. To formally assess seasonality, we examined the Autocorrelation Function (ACF) and Partial Autocorrelation Function (PACF) plots of the raw time series for significant spikes at seasonal lags (e.g. lag 12).

We then performed an ITS analysis to assess the impact of the AMS intervention on the appropriateness of carbapenem prescriptions and to determine whether the intervention led to a sustained post-intervention effect.

To account for potential autocorrelation in the time series (where the appropriateness rate in one month might be influenced by the rate in previous months), we employed an Autoregressive Integrated Moving Average (ARIMA) model with an autocorrelation error 1 (AR1). We determined the error structure to be an ARIMA (1, 0, 0) model. This specification includes an autoregressive term of order 1 (*P* = 1) to model the correlation between consecutive errors, with no differencing (*d* = 0) or moving average terms (*q* = 0) required. We compared specifications, including seasonal models, but determined that a non-seasonal ARIMA (1, 0, 0) model was the most parsimonious and appropriate, as confirmed by its residual diagnostics.

The ITS-ARIMA model included the following terms: (i) the monthly proportion of inappropriate carbapenem prescriptions as the dependent variable; (ii) a continuous variable representing time in months to model the baseline underlying trend; (iii) the immediate level change following the onset of the intervention; (iv) the trend, represented as the change in slope, following the onset of the intervention; (v) the immediate level change following the cessation of the intervention; (vi) the trend, represented as the change in slope, following the cessation of the intervention.

We estimated the baseline trend and the immediate and sustained effects for the intervention. The effect of the intervention was quantified by the estimated coefficients for these level and slope change parameters. Statistical significance for these parameters was set at *P* < 0.05.

To further illustrate the intervention’s impact, we calculated a counterfactual scenario from the fitted model. This represented the projected trend in inappropriate carbapenem prescriptions had the intervention not been implemented. We achieved this by setting the level and slope change regressors for the intervention to zero for the entire series and predicting from the model.

Finally, we conducted a *post-hoc* segmented sensitivity analysis after visual inspection of data. We introduced a new breakpoint at the series nadir (June 2023) to statistically test if this observed shape represented a true change in trend.

We conducted the statistical analyses with R software v4.2.2 and RStudio v2024.12.1 + 563 https://www.R-project.org/ [accessed on 12 May 2025].

### Ethical approvement

The study was performed following the Declaration of Helsinki and was approved by the Ethics Committee of the Fondazione Policlinico Universitario Agostino Gemelli IRCCS (reference number ID 7542).

## Results

### Descriptive analysis

Main prescriptions’ characteristics divided by pre-intervention, intervention and post-intervention phases are reported in Table 1. A total of 1825 prescriptions were evaluated: 726 in the pre-intervention period, 580 during the intervention period and 519 in the post-intervention follow-up. We observed an increase in prescriptions made by ID specialist consultants: 23% (170/726) in the pre-intervention period, rising to 37% (212/580) during the intervention and remaining stable at 34% (175/519) in the post-intervention. While non-ID physicians were the primary prescribers of carbapenems in the pre-intervention at 42% (308/726), their prescription rate in the post-intervention phase became comparable to that of ID specialist consultants, at 34% (177/519).

**Table 1.** Characteristics of carbapenem prescriptions across the study periods

Characteristic	Overall, *N* = 1,825^a^	Study period			*P*-value^b^
		Pre-intervention	Intervention	Post-intervention	
**Prescribing physician**					<0.001
ID specialist consultant	557/1825 (31)	170/726 (23)	212/580 (37)	175/519 (34)	
On-call ID specialist (written advice)	372/1825 (20)	175/726 (24)	97/580 (17)	100/519 (19)	
On-call ID specialist (phone advice)	109/1825 (6.0)	26/726 (3.6)	54/580 (9.3)	29/519 (5.6)	
Non-ID physician	671/1825 (36)	308/726 (42)	186/580 (32)	177/519 (34)	
ED doctor	116/1825 (6.4)	47/726 (6.5)	31/580 (5.3)	38/519 (7.3)	
**Reason for therapy**					<0.001
Unknown indication	37/1825 (2.0)	23/726 (3.2)	10/580 (1.7)	4/519 (0.8)	
Empiric therapy for unlikely/questionable infection	90/1825 (4.9)	48/726 (6.6)	27/580 (4.7)	15/519 (2.9)	
Empiric therapy for probable/possible infection	1060/1825 (58)	436/726 (60)	338/580 (58)	286/519 (55)	
Targeted therapy	606/1825 (33)	204/726 (28)	196/580 (34)	206/519 (40)	
Surgical prophylaxis	32/1825 (1.8)	15/726 (2.1)	9/580 (1.6)	8/519 (1.5)	
**Type of infection**					0.15
Bloodstream Infections	843/1825 (46)	318/726 (44)	289/580 (50)	236/519 (45)	
Pneumonia	252/1825 (14)	115/726 (16)	70/580 (12)	67/519 (13)	
Urinary tract infections	199/1825 (11)	78/726 (11)	66/580 (11)	55/519 (11)	
Intra-abdominal infections	278/1825 (15)	101/726 (14)	84/580 (14)	93/519 (18)	
ABSSSI	35/1825 (1.9)	20/726 (2.8)	6/580 (1.0)	9/519 (1.7)	
Osteoarticular infections	38/1825 (2.1)	15/726 (2.1)	7/580 (1.2)	16/519 (3.1)	
Surgical site infections	33/1825 (1.8)	11/726 (1.5)	16/580 (2.8)	6/519 (1.2)	
Surgical prophylaxis	31/1825 (1.7)	14/726 (1.9)	9/580 (1.6)	8/519 (1.5)	
Untraceable	60/1825 (3.3)	35/726 (4.8)	17/580 (2.9)	8/519 (1.5)	
Other	56/1825 (3.1)	19/726 (2.6)	16/580 (2.8)	21/519 (4.0)	
**Previous relevant microbiological isolations (during hospitalization)**	855/1825 (47)	252/726 (35)	343/580 (59)	260/519 (50)	<0.001
**Beta-lactams allergy**					0.45
None	1512/1825 (83)	597/726 (82)	477/580 (82)	438/519 (84)	
Reported allergy to penicillins	252/1825 (14)	95/726 (13)	85/580 (15)	72/519 (14)	
Reported allergy to cephalosporins	61/1825 (3.3)	34/726 (4.7)	18/580 (3.1)	9/519 (1.7)	
**Use of anti-Gram-negative beta-lactams in the past 90 days**	1000/1825 (55)	355/726 (49)	384/580 (66)	261/519 (50)	<0.001

ABSSI, Acute Bacterial Skin and Skin Structure Infections; ED, Emergency Department; ID, Infectious Diseases.

^a^Median (IQR) or Frequency (%).

^b^Kruskal-Wallis rank sum test.

The primary reasons for carbapenem prescription throughout the study were empiric therapy for probable/possible infection (58%, 1060/1825) and targeted therapy (33%, 606/1825). Prescriptions for targeted therapy have progressively increased from 28% (204/776) of the pre-intervention period to 40% (206/519) of post-intervention. In contrast, empiric therapy prescriptions, for both probable/possible infection and unlikely/questionable infection, decreased from 60% (436/726) to 55% (218/519) and from 6.6% (48/726) to 2.9% (15/519), respectively.

During the entire study period, the highest proportion of therapies were prescribed for suspected or confirmed bloodstream infections (46%, 843/1825).

A total of 458 inappropriate prescriptions were identified. The inappropriateness rate was 36% (260/726) in the pre-intervention period, which significantly decreased to 17% (98/580) during the intervention, and then showed a slight increase to 19% (100/519) in the post-intervention follow-up.

The main reasons for inappropriateness, as detailed in Table 2, were: too broad spectrum (72%, 331/458), failure to perform an adequate microbiological workup before starting antibiotic therapy (27%, 122/458), failure of de-escalation (20%, 92/458), and unnecessary antibiotic therapy (16%, 71/458).

**Table 2.** Reasons for inappropriate carbapenem prescriptions across study periods

Reason for inappropriate prescribing	Overall, *N* = 458^a^	Study period			*P*-value^b^
		Pre-intervention	Intervention	Post-intervention	
Unnecessarily broad spectrum	331/458 (72)	187/260 (72)	59/98 (60)	85/100 (85)	<0.001
Adequate microbiological workup not performed	122/458 (27)	68/260 (26)	34/98 (35)	20/100 (20)	0.065
Unnecessary antibiotic therapy	71/458 (16)	44/260 (17)	16/98 (16)	11/100 (11)	0.38
Failure of de-escalation	92/458 (20)	51/260 (20)	24/98 (24)	17/100 (17)	0.42
Incorrect antibiotic dosage	8/458 (1.7)	2/260 (0.8)	6/98 (6.1)	0/100 (0)	0.003
Excessive treatment duration	26/458 (5.7)	15/260 (5.8)	5/98 (5.1)	6/100 (6.0)	>0.99
Escalation not indicated	13/458 (2.8)	12/260 (4.6)	0/98 (0)	1/100 (1.0)	0.035
Inappropriate or not coded prophylaxis	16/458 (3.5)	8/260 (3.1)	3/98 (3.1)	5/100 (5.0)	0.61

^a^Median (IQR) or Frequency (%).

^b^Fisher's exact test.

Prescribing physicians responsible for inappropriate carbapenem prescriptions are reported in Table 3. The majority of inappropriate prescriptions (338/458) were issued by non-ID physicians, maintaining a consistent rate across all three observation periods. Inappropriate prescriptions by ID specialist consultants decreased from 5.4% (14/260) in the pre-intervention period to 3% (3/100) in the post-intervention follow-up. Similarly, those by on-call ID specialists with written advice decreased from 10% (27/260) in the pre-intervention to 3% (3/100) in the post-intervention.

**Table 3.** Distribution of inappropriate carbapenem prescriptions by prescriber across the study periods

Prescribing physician	Overall, *N* = 458^a^	Study period			*P*-value^b^
		Pre-intervention	Intervention	Post-intervention	
					0.09
ID specialist consultant	25/458 (5.5)	14/260 (5.4)	8/98 (8.2)	3/100 (3.0)	
On-call ID specialist (written advice)	38/458 (8.3)	27/260 (10)	8/98 (8.2)	3/100 (3.0)	
On-call ID specialist (phone advice)	18/458 (3.9)	11/260 (4.2)	3/98 (3.1)	4/100 (4.0)	
Non-ID physician	338/458 (74)	189/260 (73)	73/98 (74)	76/100 (76)	
ED doctor	39/458 (8.5)	19/260 (7.3)	6/98 (6.1)	14/100 (14)	

ED, Emergency Department; ID, Infectious Diseases.

^a^Median (IQR) or Frequency (%).

^b^Fisher's exact test.

Subgroup analyses, as detailed in Table 4, showed that inappropriate prescriptions were more frequent among empiric therapies compared with targeted therapies across all study periods. Specifically, inappropriate empiric prescriptions decreased from 39% (190/484) in the pre-intervention to 19% (70/365) during the intervention, then slightly increased to 24% (73/301) in the post-intervention; while inappropriate targeted prescriptions were consistently lower: 18% (36/204) pre, 7% (14/196) during, 8% (17/206) post the intervention.

**Table 4.** Inappropriateness rates by therapy type and infection source across study periods

Characteristic	Overall^a^	Study period			*P*-value^b^
		Pre-intervention	Intervention	Post-intervention	
**Reason for therapy**					<0.001
Empiric therapy	333/1150 (29)	190/484 (39)	70/365 (19)	73/301 (24)	
Targeted therapy	67/606 (11)	36/204 (18)	14/196 (7)	17/206 (8)	
**Type of infection**					
Bloodstream Infections	145/843 (17)	86/318 (27)	33/289 (11)	26/236 (11)	
Pneumonia	69/252 (27)	41/115 (36)	11/70 (16)	17/67 (25)	
Urinary tract infections	55/189 (29)	29/78 (15)	10/66 (29)	16/55 (29)	
Intra-abdominal infections	69/278 (25)	36/101 (36)	17/84 (20)	16/93 (17)	

^a^Median (IQR) or Frequency (%).

^b^Fisher's exact test.

When stratified by infection source, the highest rates of inappropriate prescribing were observed for pneumonia and intra-abdominal infections. All infection types showed a reduction in inappropriate use during the intervention phase, with a partial rebound post-intervention. Bloodstream infections had the lowest inappropriate rates throughout the study period (data are shown on Table 4).

### Interrupted time series analysis

The final ITS model was fitted using an ARIMA (1, 0, 0) error structure with the ITS variables included as exogenous regressors. The model's fit was assessed using standard diagnostics. The Akaike Information Criterion was 58.8 and the Bayesian Information Criterion was 49.8.

The model's residuals were examined for evidence of remaining autocorrelation. The Ljung-Box Q-test statistic confirmed the adequacy of the ARIMA (1, 0, 0) specification, yielding a *P*-value of 0.40. Since *P* > 0.05, this suggests that the residuals are statistically indistinguishable from white noise, meaning the model successfully accounted for the serial correlation in the time series. Furthermore, visual inspection of the ACF and PACF plots of the residuals (Supplementary material, available as Supplementary data at *JAC-AMR* Online) showed that nearly all spikes fell within the 95% confidence bounds, reinforcing that the error structure was appropriately specified. No significant spikes were observed at the seasonal lag of 12, confirming that a seasonal component was not necessary for the final model.

**Figure 2.**
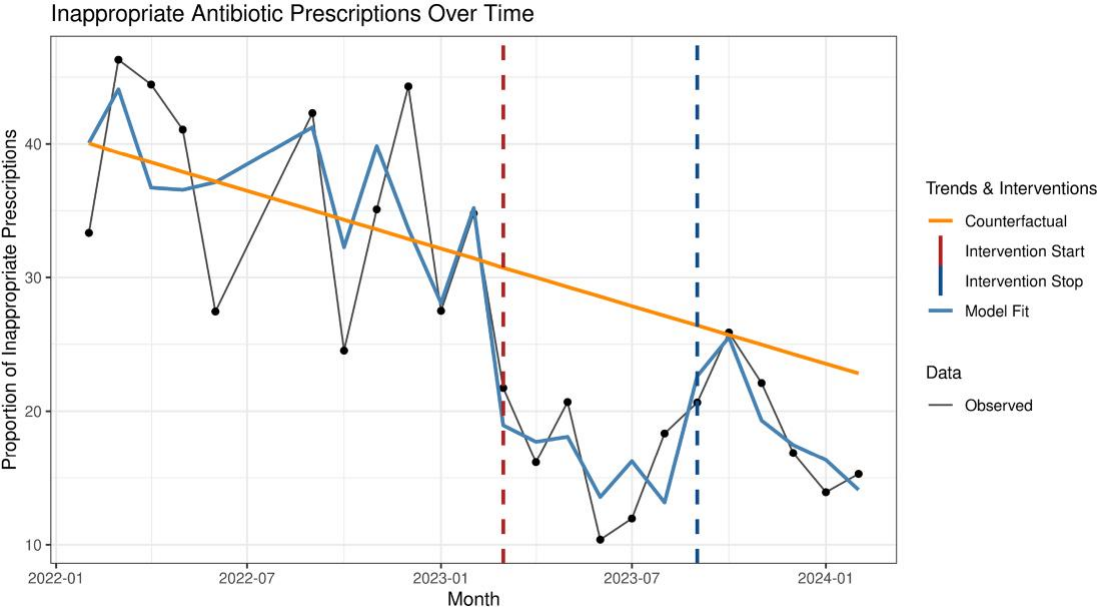
Interrupted Time Series Analysis of Inappropriate Carbapenem Prescriptions Using ARIMA Model.

The ITS-ARIMA model (Figure 2) revealed an immediate and statistically significant 11% decrease in the rate of inappropriateness of carbapenem prescriptions (*P* = 0.001) following the intervention’s onset. In the counterfactual scenario, the proportion of inappropriateness was estimated to decrease by 7%, before the intervention occurred, holding others factors constant. After the implementation of the intervention, the monthly trend of inappropriateness changed by −8% per month (*P* = 0.340), in addition to the baseline trend of the time-point. In the first month of the post-intervention period, there was an immediate and statistically significant 14.9% increase in the rate of inappropriateness (*P* = 0.001), compared with what would have been expected based on the established post-intervention. In the post-intervention follow-up, the monthly trend of inappropriateness changed by −9% per month. However, similar to the intervention phase, this change in slope was not statistically significant (*P* = 0.443).

For the sensitivity *post hoc* analysis, we used a new breakpoint at the nadir of inappropriate rates (June 2023). The model estimated a non-significant increase of 3.7% points per month (*P* = 0.33). This finding suggests the observed rebound was likely due to random variation rather than a structural change in the trend.

## Discussion

In this retrospective study, applying an ITS analysis, we documented a significant and immediate reduction in the rate of inappropriate carbapenem prescriptions during the post-prescription audit intervention. We also observed a reduction in the trend of inappropriateness, although this change was not statistically significant. This immediate and significant impact may suggest the effectiveness of a post-prescription audit intervention in reducing inappropriateness. It may also reflect the influence of broader initiatives associated with our hospital’s Joint Commission International Accreditation process (e.g. complementary measures implemented by the internal Pharmacy Department), which may have indirectly reinforced AMS awareness and behaviours among prescribers. Conversely, we reported an immediate and statistically significant increase in the rate of inappropriateness during the first month of the post-intervention period, indicating a potential lack of sustained effectiveness once the audit ceased.

Several studies employing ITS analysis evaluate the impact of AMS strategies on antibiotic prescription.^21–23^ These studies often report immediate effects after intervention implementation, with varying degrees of long-term sustainability. For instance, a cross-over trial conducted in the United States demonstrated that prospective audit and feedback is more effective than prior authorization in reducing antibiotic use, as measured by days of antibiotic therapy.^24^

A key strength of our study is the choice of antibiotic appropriateness as the primary measure of AMS intervention effectiveness, rather than antibiotic consumption, which is typically used in similar studies.^25–27^ However, a limitation of using appropriateness is the lack of a universally standardized definition. To achieve the most objective assessment possible, we utilized an algorithm to define antibiotic therapy inappropriateness, which specified the reasons for inappropriateness and referenced approved national, international and local guidelines.

The observed reduction in inappropriate carbapenem prescriptions by both ID specialist consultants and on-call ID specialists (with written advice), can be attributed to the regular audits organized with these prescribing ID specialists. These audits provided them with the opportunities for learning through comparison of individual prescriptions with their ID colleagues. Furthermore, their awareness of being ‘observed’ throughout the intervention period likely contributed to a Hawthorne effect.^28^ Considering the increased proportion of prescriptions made by ID specialists during both the intervention and post-intervention phases, it is plausible that the combination of face-to-face feedback and heightened oversight increased prescribers’ awareness and encouraged earlier involvement of ID consultants. However, this potential behavioural change cannot be quantified within this study and should be interpreted cautiously.

Regarding the reasons for inappropriateness, ‘too broad spectrum’ consistently remained the main cause, even in the post-intervention phase. This finding suggests that the relatively short duration of the intervention, the reliance on prospective audits alone, and the *ad personam* nature of the feedback (rather than broader unit-wide educational meetings) may be insufficient to fully instill principles of adequate empirical therapy.^29^

These results, particularly the immediate reduction in inappropriate carbapenem use, may suggest potential practical applications for clinical AMS. Hypothetically, the consistent implementation of structured post-prescription audits, especially when supported by dedicated ID specialist involvement, could serve as a powerful tool to drive immediate and substantial improvements in prescribing quality within healthcare institutions. However, the temporary nature of the effect highlights that sustained impact likely requires continuous or strategically pulsed interventions rather than one-off campaigns.

This study presents several limitations. First, all assessments of prescribing appropriateness in pre and post-intervention phases were conducted retrospectively. Despite the use of a standardized, algorithm-based evaluation, this retrospective approach may have introduced classification bias. Second, although the algorithm was rigorously developed using international, national, and institutional guidelines, it has not yet undergone formal or external validation. Nonetheless, its structure was adapted from validated frameworks such as the Hospital NAPS, which supports its conceptual soundness and applicability within stewardship practice. Third, while the ITS design strengthens causal inference in quasi-experimental settings, it cannot fully account for unmeasured confounders or simultaneous interventions that may have influenced prescribing patterns. Although the study covered two full seasonal cycles and no concurrent stewardship programs were implemented, the potential influence of contextual factors related to the Joint Commission International Accreditation process cannot be entirely excluded. Fourth, the study was conducted at a single academic tertiary care centre, which may limit the generalizability of the findings to other settings with different prescribing cultures or resources. Future specific studies, with more robust and generalizable study designs, are needed to determine the duration of the effect on prescription appropriateness and sustainability of various stewardship interventions across different healthcare settings and resource levels. Key methodological advancements should include prospective studies that incorporate independent evaluation of appropriateness and rigorous external validation of appropriateness algorithms across different institutions to enhance reproducibility. Furthermore, future investigations should consider and collect detailed data on potential confounders, including co-interventions and patient-level factors, to strengthen causal inference. The absence of a sustained effect after the intervention suggests that the behavioural and prescribing improvements achieved through direct, real-time feedback may diminish once active reinforcement ceases. This finding underscores the importance of designing stewardship programs that incorporate continuous or periodic feedback mechanisms, integration into routine clinical workflows, and institutional commitment to long-term monitoring. In this sense, the short-term impact observed in our study provides valuable insight into how stewardship interventions may need to evolve from project-based initiatives to permanent components of clinical governance.

### Conclusion

The described AMS intervention proved effective in reducing the rate of inappropriate carbapenem prescriptions, demonstrating an immediate impact that was, however, not sustained over the subsequent 6 months. In the absence of a standardized definition of therapy appropriateness, we believe that the proposed IATTURA method warrants further evaluation. Methodologically robust studies, particularly those employing designs such as ITS analyses, are increasingly important to evaluate the impact of various AMS interventions on antibiotic appropriateness, especially when assessed at multiple time points before and after the intervention. Such studies are essential for planning AMS strategies that are sustainable in terms of both human resources and time investment.

## Supplementary Material

dlaf236_Supplementary_Data

## Supplementary data

Figure S1 and S2 available as Supplementary data at *JAC-AMR* Online.
