# Supplementary material for: Impact of a post-prescription audit and feedback antimicrobial stewardship intervention on inappropriate carbapenem prescribing: an interrupted time series analysis
Source: JAC Antimicrob Resist. 2025 Dec 3;7(6):dlaf236. doi: 10.1093/jacamr/dlaf236 (PMC12673262; doi:10.1093/jacamr/dlaf236)
Supplement: dlaf236_Supplementary_Data [file dlaf236_supplementary_data.docx]

**Supplementary material**

**Figure S1:** Autocorrelation function (ACF) plot**
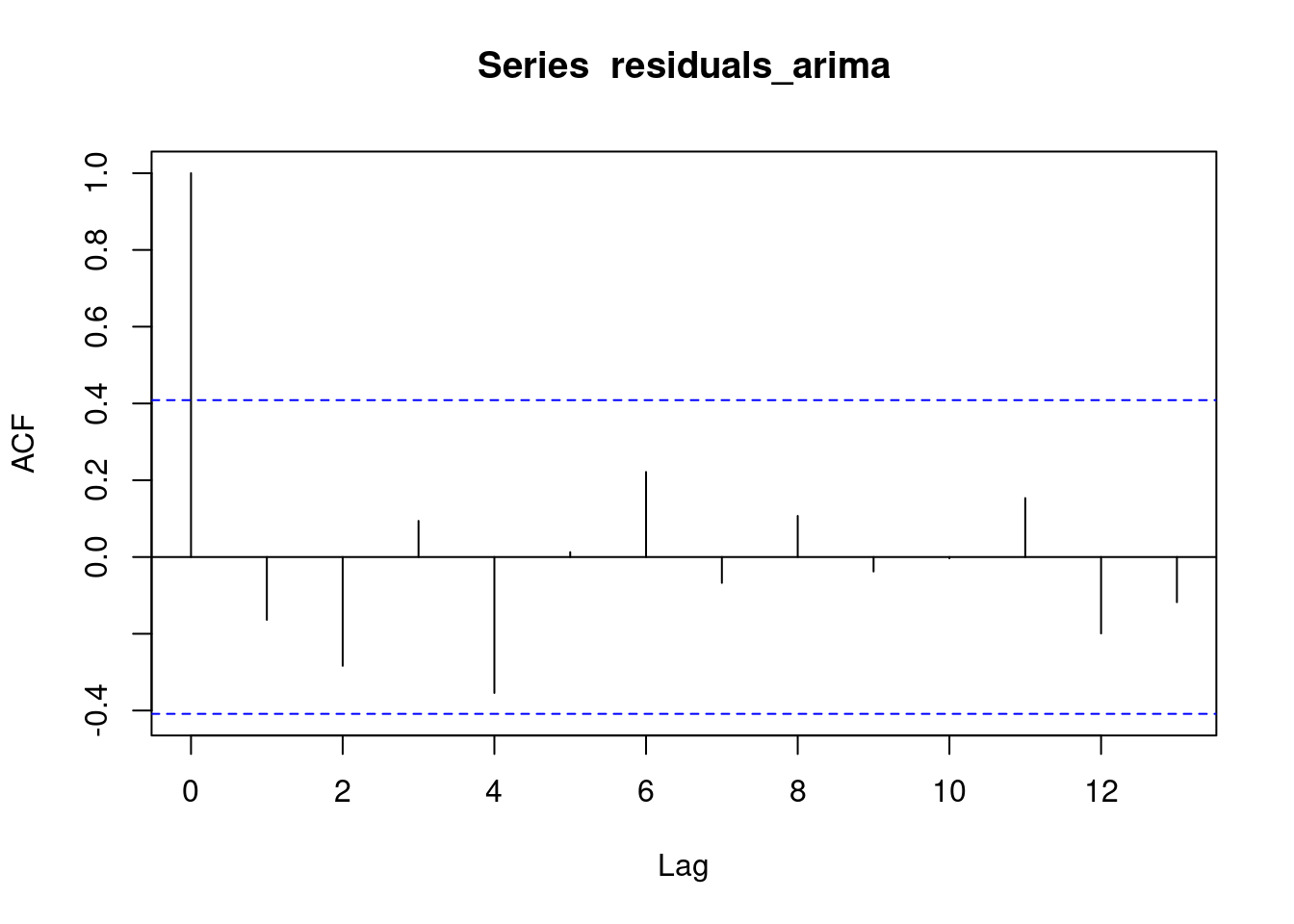
**

Figure S2: Partial Autocorrelation function (PACF) plot

**
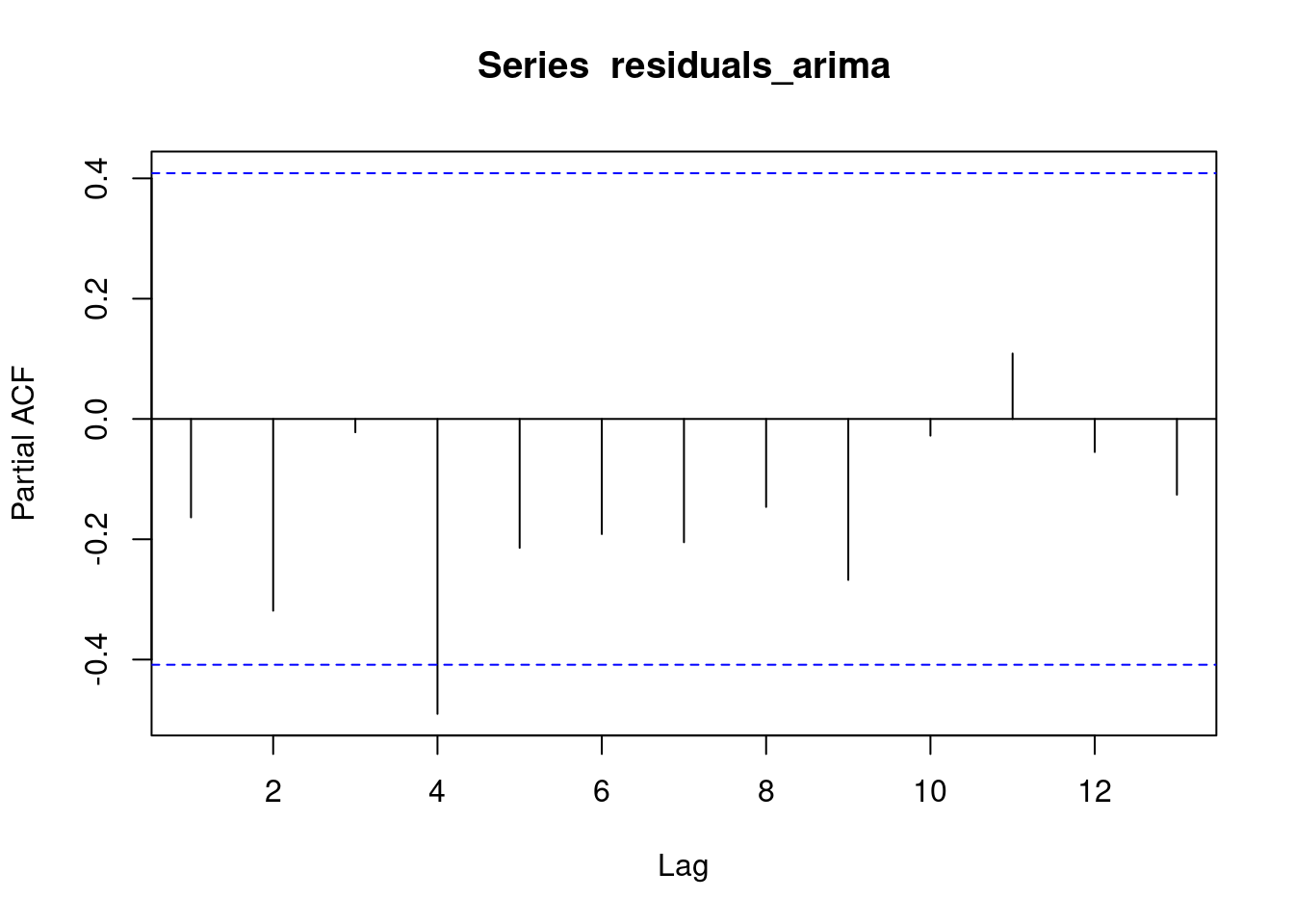
**
